# Healthcare Affordability and Associated Concerns Among Older Adults in Florida

**DOI:** 10.1093/geroni/igaa057.321

**Published:** 2020-12-16

**Authors:** Jacqueline Wiltshire, Barbara Orban, Kyaien Conner, Edlin Garcia Colato, Erica Anderson, Iraida Carrion

**Affiliations:** University of South Florida, Tampa, Florida, United States

## Abstract

Rising healthcare costs create significant financial burden for Americans and is a threat to the well-being of our growing, racially/ethnically diverse, older population. In a mixed method study, we assessed ability to afford care and ascertain concerns about healthcare cost in a racially diverse sample of Floridians ages ≥ 65. We surveyed 170 adults (40.4% White, 27.6% African Americans/Black and 31.8% Latino/Hispanic) and conducted three race-stratified focus groups (n=27). Most participants had Medicare coverage (97.1%) and 27% also had Medicaid. Approximately 11.6% of Whites had problems paying medical bills in the past 12 months versus 14.9% of African Americans/Blacks and 24.1% of Latino/Hispanics. Additionally, 13% of Whites, 19.2% of African Americans/Blacks and 20.4% of Hispanics reported not getting needed prescription drugs because they could not afford them. Approximately 45.7% either perceived that their doctor “never” takes into account costs for treatment or did not know whether costs were considered. Multiple regression analyses showed no statistically significant racial/ethnic differences in affordability problems. From the focus groups, healthcare cost concerns most frequently identified by participants were the high cost of prescriptions drug, rising co-pays and out of pocket expenses, and medical billing. Participants’ concerns about medical billing included understanding their bills, transparency in billing, timely posting of charges, and uncertainty about who to talk to about billing problems. Our findings suggest that routine discussions about healthcare costs with doctors or designated healthcare personnel should help ease financial burden and healthcare costs concerns among older adults.

